# Post-Partum and Other Hemorrhages

**Published:** 1888-06

**Authors:** A. B. Eadie


W. J. Hunter Emory,

A. B. Eadie.

---

### POST-PARTUM AND OTHER HEMORRHAGES.

### PLATINA.

*Characteristics.*

1. Apprehensive ; fears about recovery, coupled with fault-finding ; lachrymose, or merry, unnatural liveliness, ending in haughtiness and contempt of others.
2. Pains increase and decrease slowly and gradually.
3. During sleep lies on back ; arms over head ; thighs flexed and legs uncovered.

*Special indications.*

Menses too early, copious, and short lasting.

Metrorrhagia, blood very dark, thick, and tarry; not coagulated in masses; with pains in small of back and sacrum; pressing pains, as if the parts would come out; great sensitiveness of the external organs; labor pains, interrupted by sensitiveness of vagina and vulva ; after labor so sensitive she cannot bear touch of napkin.

Nymphomania; feels as if the limbs and body were growing large.

*Generalities.*

Constipation; stool seems to adhere to anus like putty ; worse traveling ; dark-haired females, *rigid fibres ;* great inclination to violent, spasmodic yawning ; worse in evening; worse from anger; *worse when lying down* and while sitting, from tapeworm.

Restlessness of body ; worse on resting ; better moving ; better on and *after* rising from bed or *seat;* better walking.

### SULPHURIC ACID.

*General characteristics.*

1. Great exhaustion.
2. Sensation of tremor all over without actual trembling.
3. Pains gradually coming, suddenly ceasing (come and go quickly—Bell.; come and go gradually—Stann., Plat.)
4. Impatience. Nobody does anything quick enough for her. (Cham.)

*Special indications.*

Hemorrhages of black blood from all the outlets of the body.

Menses too early and too profuse, and preceded by distressing nightmare.

Oozing of dark, thin blood.

*Generalities.*

Aversion to water; it chills the stomach unless mixed with spirit.

In injuries from being bruised or cut, especially when ecchimosed, it competes with Arnica, from which it is differentiated as follows:

| ARNICA. | SULPH. ACID. |
|---|---|
| Oversensitiveness to pain; | Lacks oversensitiveness; |
| worse from being overhurried; | can't do things quick enough; |
| better in open air; | worse in open air; |
| worse in-doors. | better in room. |

### STRAMONIUM.

*Characteristics.*

1. Delirium, loquacity, hallucinations, talks all the time, sings, makes verses; hallucinations terrify the patient; sees ghosts, bugs, or beasts running at her.
2. Aversion to water; thirst, but inability to swallow; unable to speak.
3. Urine suppressed with urging; constipation, with desire for stool.

*Special indications.*

Metrorrhagia, loquacity, singing, praying, sometimes passing large coagula.

*Special indications.*

Threatened abortion, unceasing talking, singing, imploring. Excessive menstrual flow.

Menstrual flow watery; during menses loquacity and strong smell as of semen.

Nymphomania, lewd talking.

*Generalities.*

Arms agitated, lower limbs quiet.

Covers up during heat; pupils dilated.

Vision affected; fog before eyes; can't distinguish colors; double vision; shuns light but yet likes room illumined. Aversion to water; sight of it causes spasms; can't drink, or if he can does so in a spasmodic manner; worse during perspiration; worse after sleep; worse from spirits. (Contra Sulph-ac.)

A. B. EADIE.

---

### CHAMOMILLA.

The grand characteristics of Chamomilla are the mental and general disposition of the patient; it is suited to hateful, vindictive natures (Nitr-ac., Calc., Natr-mur.); patient *cannot bear any one near and answers snappishly.* She becomes angry and almost furious about the pain; great impatience, everything seems to go too slowly (Sulph-ac.). Patient has frequent pressure toward uterus, like labor pains; sensation as if sacrum were being separated; frequent desire to urinate, large quantities of pale urine. Reflected tearing pains in the legs.

The hemorrhages may be convulsive (*Hyos.*, *Ipecac.*, Platina, Cinnam., Secale), intermitting (Puls.); there is profuse discharge of clotted blood with severe labor-like pains.

Metrorrhagia, even in old women, of dark, clotted blood, flowing mostly in paroxysms (black blood in a *stream*, Kreos.); much thirst; coldness of limbs. Excessive flow of dark, nearly black, *clotted*, fœtid blood (black, *liquid* fœtid, Secale), attacks of syncope. Uterine hemorrhages after abortion, in spells; contracting, out-pressing pains from back to front; worse lying in bed. Post-partum flow; through abdominal walls uterus can be felt to be contracted into little knots about the size of a walnut; redness and heat of one cheek (left) with other pale and cold (also Arn., Ignatia; red cheek cold, pale one hot, Mosch.).

### APIS MELLIFICA.

Apis mellifica is especially adapted to flooding or hemorrhages brought on or sustained by hatred or jealousy; there are usually stinging pains and great sensitiveness over abdominal tract, especially right ovary (left Vespa). Metrorrhagia, with heaviness in abdomen, syncope, restlessness, yawning, and there may be red spots like bee stings on body.

Abortion during early months, stinging pains till labor pains ensue; scanty urine, *no* thirst (reverse Cham.); profuse flow. There may be bag-like swelling *under* eyes (Kali carb., *over* eyes); general aggravation from heat, likes to kick off covers (*Secale*); pains are ameliorated by cold water (also Fluoric ac.; reverse Sil.). J. D. TYRRELL.

---

### ARNICA MONTANA.

In all hemorrhages which can be traced to traumatism of any kind, such as severe falls, blows, bruises, etc., etc., Arnica is the first remedy to be thought of and should always be administered, unless symptoms call strongly for some other remedy.

This will also apply to hemorrhages following severe, protracted labors and instrumental deliveries.

Hemorrhages bright red or mixed with clots; head hot and body cool.

When patient complains of sore, bruised sensations.

Also in hemorrhages following coition.

### APOCYNUM CANNABINUM.

This remedy seems more likely to be indicated in metrorrhagias than in post-partum hemorrhages.

Very profuse flow of blood, preceded for a day or two by a moderate discharge.

Hemorrhage ceasing at intervals; always recurred when the vital powers rallied.

Blood expelled in large clots, sometimes in a fluid state.

Sudden, profuse hemorrhages during catamenia; shreds or pieces of membrane with the fluid blood.

*Metrorrhagia continuous or paroxysmal; fluid or clotted;* nausea, vomiting, palpitation; pulse quick and feeble when moved; fainting when raising the head from the pillow.

From a cold during a sleigh ride, after the catamenia had set in, menorrhagia lasting four and a-half weeks.

### BRYONIA ALBA.

*The hemorrhage is dark red and increased by the slightest movement of any part of the body, even the hand or foot or speaking;* also Sabina and Crocus sat. Crocus, however, has the *dark or black stringy flow.* Adapted to brunettes, dark skin, hair, and eyes, tall and slim, with firm, fleshy fibre.

W. J. HUNTER EMORY.

---

### HYOSCYAMUS.

Hyoscyamus will chiefly be found indicated in post-partum and other accidental hemorrhages in women inclined to obesity, with lax muscles and skin; to hysterical subjects, nervous, irritable, excitable, and of a sanguine temperament.

Almost invariably the brain and nerves indicate a high state of tension, oppression, or irritability. Accordingly, we commonly find a tendency to spasms and convulsions in connection with post-partum hemorrhage when this drug is the simillimum.

In fact, in my experience, the tendency to convulsions is in these cases much more alarming than the hemorrhage, which is usually a *continuous, bright red flow,* but occasionally, when convulsions are actually present, a *pale flow.*

In all cases we find a great vascular excitement.

Briefly, then, where Hyos. is indicated we shall find fullness of the veins, full pulse, and hemorrhages usually light red.

As very pertinent to this subject, I call your attention to the fact that this drug, better than any other of which I know, overcomes the ill effects induced by the inhalation of ether.

### HELONIAS DIOICA.

In cases to which this remedy is suitable we find the mind dull, gloomy, and inactive, together with a high degree of irritability, tendency to fault-finding, and a marked desire to be let severely alone.

It will generally be found useful in cases of marked general debility, lack of tone, or anæmia. I will merely suggest its probable usefulness in such lying-in patients as have suffered from albuminaria throughout their pregnancy, also to such as have been martyrs to the long list of so-called "female diseases."

In hemorrhages, occurring in these thoroughly broken-down subjects, in whom from lack of vitality the uterus seems to have lost its contractile power and where the flow is passive, dark, coagulated, and possibly offensive, we shall probably find Helonias most useful.

Unfortunately, as yet, this drug has not received the thorough "proving" it deserves. So far as is now known, it gives promise of untold blessings to a suffering womanhood.

### HAMAMELIS.

Hamamelis is indicated in the mentally depressed, exhausted, and irritable.

It is adapted to those exhibiting a marked tendency to hemorrhage, and in whom a small loss of blood causes a great amount of prostration. Much weariness; complains of being *so tired.*

In uterine hemorrhage the *flow is steady* and slow; the blood is dark colored (venous). Absence of uterine pains; profuse discharge of dark blood.

I may be permitted to mention a characteristic symptom of this drug in general uterine hemorrhage, as it did me good service in a trying case, viz.: the flow occurs only in daytime, ceases at night. (Compare *Causticum* and Pulsatilla.)

Briefly, then, we shall do well to think of this remedy in patients exhibiting a hemorrhagic diathesis and where we find a passive venous flow.

EDWARD ADAMS.

---

### ACONITUM.

This remedy is specially suitable to those of full habit and firm fibre. The flow is active, the patient restless, anxious, and fears she will die. Activity and force seem to be the key-notes for this drug, it being adapted generally to the early stages of any disorder, before the powers are exhausted, or the fluids or tissues are in any way disorganized. Aconitum would be probably more used than any other medicine were it not that in a pretty large number of cases the time for its usefulness is past and gone before we are called to see the patient. It is hardly necessary to add, that a frequent pulse, strong and full, or, if contracted, still strong and tense, is a strong indication for this remedy.

### CAULOPHYLLUM

is chiefly indicated after a hasty labor; the child seems to have been expelled too soon, so that the womb loses the pressure of its contents before the time has arrived at which its own contractive forces will suffice for stanching the flow. The premature expulsion is apparently due to weakness rather than force, or rather to a want of proper co-ordination of the contractive powers of the uterine zones, the cervical fibres being proportionally more relaxed than those of the fundus, the very opposite condition from that which prevails in some cases calling for Belladonna.

### ERIGERIA

seems to be more frequently called for in menorrhagia than in true hemorrhages. When indicated in the latter, the flow is profuse and bright red, increased by the slightest motion, kept in check to a certain extent by remaining perfectly still. The patient is pale and weak from the excessive flow, while irritation of the bladder and rectum combine to aggravate her distress. HAMILTON EVANS, M. D.

